# Supplementary material for: A fault-intrusion-tolerant system and deadline-aware algorithm for scheduling scientific workflow in the cloud
Source: PeerJ Comput Sci. 2021 Nov 2;7:e747. doi: 10.7717/peerj-cs.747 (PMC8576554; doi:10.7717/peerj-cs.747)
Supplement: Supplemental Information 6 [file peerj-cs-07-747-s006.zip › Myproject1.1/docs/org/cloudbus/cloudsim/power/class-use/PowerVmAllocationPolicyAbstract.html]

Uses of Class org.cloudbus.cloudsim.power.PowerVmAllocationPolicyAbstract


JavaScript is disabled on your browser.


- Overview
- Package
- Class
- Use
- Tree
- Deprecated
- Index
- Help

- Prev
- Next

- Frames
- No Frames

- All Classes

## Uses of Class org.cloudbus.cloudsim.power.PowerVmAllocationPolicyAbstract

- Packages that use PowerVmAllocationPolicyAbstract

  | Package | Description |
  | --- | --- |
  |  |  |
  | --- | --- |
  | org.cloudbus.cloudsim.power |  |
- - ### Uses of PowerVmAllocationPolicyAbstract in org.cloudbus.cloudsim.power

    Subclasses of PowerVmAllocationPolicyAbstract in org.cloudbus.cloudsim.power

    | Modifier and Type | Class and Description |
    | --- | --- |
    |  |  |
    | --- | --- |
    | `class` | `PowerVmAllocationPolicyMigrationAbstract` The class of an abstract power-aware VM allocation policy that dynamically optimizes the VM allocation using migration. |
    | `class` | `PowerVmAllocationPolicyMigrationInterQuartileRange` The Inter Quartile Range (IQR) VM allocation policy. |
    | `class` | `PowerVmAllocationPolicyMigrationLocalRegression` The Local Regression (LR) VM allocation policy. |
    | `class` | `PowerVmAllocationPolicyMigrationLocalRegressionRobust` The Local Regression Robust (LRR) VM allocation policy. |
    | `class` | `PowerVmAllocationPolicyMigrationMedianAbsoluteDeviation` The Median Absolute Deviation (MAD) VM allocation policy. |
    | `class` | `PowerVmAllocationPolicyMigrationStaticThreshold` The Static Threshold (THR) VM allocation policy. |
    | `class` | `PowerVmAllocationPolicySimple` This a simple class representing a simple VM allocation policy that does not perform any optimization of the VM allocation. |

- Overview
- Package
- Class
- Use
- Tree
- Deprecated
- Index
- Help

- Prev
- Next

- Frames
- No Frames

- All Classes
